# Single-cell reconstruction of differentiation trajectory reveals a critical role of ETS1 in human cardiac lineage commitment

**DOI:** 10.1186/s12915-019-0709-6

**Published:** 2019-11-13

**Authors:** Hang Ruan, Yingnan Liao, Zongna Ren, Lin Mao, Fang Yao, Peng Yu, Youqiong Ye, Zhao Zhang, Shengli Li, Hanshi Xu, Jiewei Liu, Lixia Diao, Bingying Zhou, Leng Han, Li Wang

**Affiliations:** 1 State Key Laboratory of Cardiovascular Disease, Fuwai Hospital, National Center for Cardiovascular Diseases, Chinese Academy of Medical Sciences and Peking Union Medical College, 167 North Lishi Road, Beijing, 100037 People’s Republic of China; 2 Department of Biochemistry and Molecular Biology, The University of Texas Health Science Center at Houston McGovern Medical School, 6431 Fannin St, MSB6.166, Houston, TX 77030 USA; 3 Department of Bioinformatics and Computational Biology, University of Texas MD Anderson Cancer Center, Houston, TX 77030 USA

**Keywords:** Cardiac lineage commitment, Human pluripotent stem cells, Single-cell RNA sequencing, Cell-cell crosstalk, ETS1, Transcription regulation

## Abstract

**Background:**

Cardiac differentiation from human pluripotent stem cells provides a unique opportunity to study human heart development in vitro and offers a potential cell source for cardiac regeneration. Compared to the large body of studies investigating cardiac maturation and cardiomyocyte subtype-specific induction, molecular events underlying cardiac lineage commitment from pluripotent stem cells at early stage remain poorly characterized.

**Results:**

In order to uncover key molecular events and regulators controlling cardiac lineage commitment from a pluripotent state during differentiation, we performed single-cell RNA-Seq sequencing and obtained high-quality data for 6879 cells collected from 6 stages during cardiac differentiation from human embryonic stem cells and identified multiple cell subpopulations with distinct molecular features. Through constructing developmental trajectory of cardiac differentiation and putative ligand-receptor interactions, we revealed crosstalk between cardiac progenitor cells and endoderm cells, which could potentially provide a cellular microenvironment supporting cardiac lineage commitment at day 5. In addition, computational analyses of single-cell RNA-Seq data unveiled ETS1 (ETS Proto-Oncogene 1) activation as an important downstream event induced by crosstalk between cardiac progenitor cells and endoderm cells. Consistent with the findings from single-cell analysis, chromatin immunoprecipitation followed by high-throughput sequencing (ChIP-Seq) against ETS1 revealed genomic occupancy of ETS1 at cardiac structural genes at day 9 and day 14, whereas ETS1 depletion dramatically compromised cardiac differentiation.

**Conclusion:**

Together, our study not only characterized the molecular features of different cell types and identified ETS1 as a crucial factor induced by cell-cell crosstalk contributing to cardiac lineage commitment from a pluripotent state, but may also have important implications for understanding human heart development at early embryonic stage, as well as directed manipulation of cardiac differentiation in regenerative medicine.

## Introduction

Differentiation of human embryonic stem cell (hESC) into cardiomyocytes (CMs) has been an essential model system to provide insights into the molecular mechanism of heart development [1–4]. Derived CMs are a powerful tool for modeling cardiovascular diseases and drug toxicity screens and also gain widespread attention in heart regeneration [5]. Recently, several studies used human pluripotent stem cell-derived cardiomyocytes or cardiac progenitors to repair injured myocardium in primates or even in humans [6–8]. These studies displayed considerable remuscularization and improved cardiac function, which shed light on the application of pluripotent stem cells in heart regenerative medicine. Despite these remarkable advances, adverse effects, such as arrhythmia and teratoma formation, still impede successful clinical translation, highlighting the need for a deeper understanding of the molecular paths from pluripotent stem cells to cardiomyocytes.

Compared to our knowledge of CM subtype specification and maturation at later stages of differentiation [4, 9, 10], much less is known about cardiac fate commitment at earlier stages. This was partially due to adverse effects observed in translational studies, which raised cell maturation and subtype specification as more important questions for regeneration purposes. For instance, a recent study achieved better maturation of CMs by starting physical conditioning early in the differentiation process and increasing its intensity over time [9]. Other fronts of the protocol improvement include developing techniques to induce specific cardiomyocyte subtypes, as well as identifying early markers of cell fate prediction during differentiation [10]. By contrast, early cardiac cell fate commitment, a critical step during heart development, is less well understood, and thus is worth exploiting for pivotal regulators of cardiac fate decisions.

Using bulk RNA-Seq technology, temporal profiling of transcriptomes during in vitro hESCs to CMs differentiation had been useful to reveal the overall dynamics of gene expression during cardiac development [1, 11]. Nevertheless, a variety of cells at different development states or from distinctly differentiated fates are mixed when performing bulk RNA-Seq, obscuring potential critical molecular events and signals taking place in cell subpopulations. Recent advancements in single-cell sequencing technology enable high-resolution observation of in vitro cardiac differentiation from hESCs to CMs dynamically [2, 4]. While cellular heterogeneity and transcriptional networks were explored in these studies, the main focus was still on possible later-stage mechanisms that lead to the immaturity of derived CMs. Therefore, to complement the missing piece of the molecular basis of cardiac lineage commitment at an early stage, we performed single-cell RNA sequencing (scRNA-Seq) across six key time points (days 0, 2, 5, 9, 14, 60) in the process of in vitro hESC-to-CM differentiation. We applied computational methods to analyze heterogeneity, to reconstruct differential trajectories, and to explore the transcription factor network around the pivotal stages of cardiac fate commitment. We demonstrated the essential role of side populations in providing a cellular microenvironment for cardiac development. We also reported the key regulatory role of the ETS1 of transcription factors in cardiac lineage specification, based on computational analysis and experimental validation. In addition, we also created an online resource for visualizing our Single-Cell data (https://hanlab.uth.edu/shiny/hESC2CM/).

## Results

### Comprehensive analysis of cardiac differentiation at single-cell resolution

**Fig. 1:**
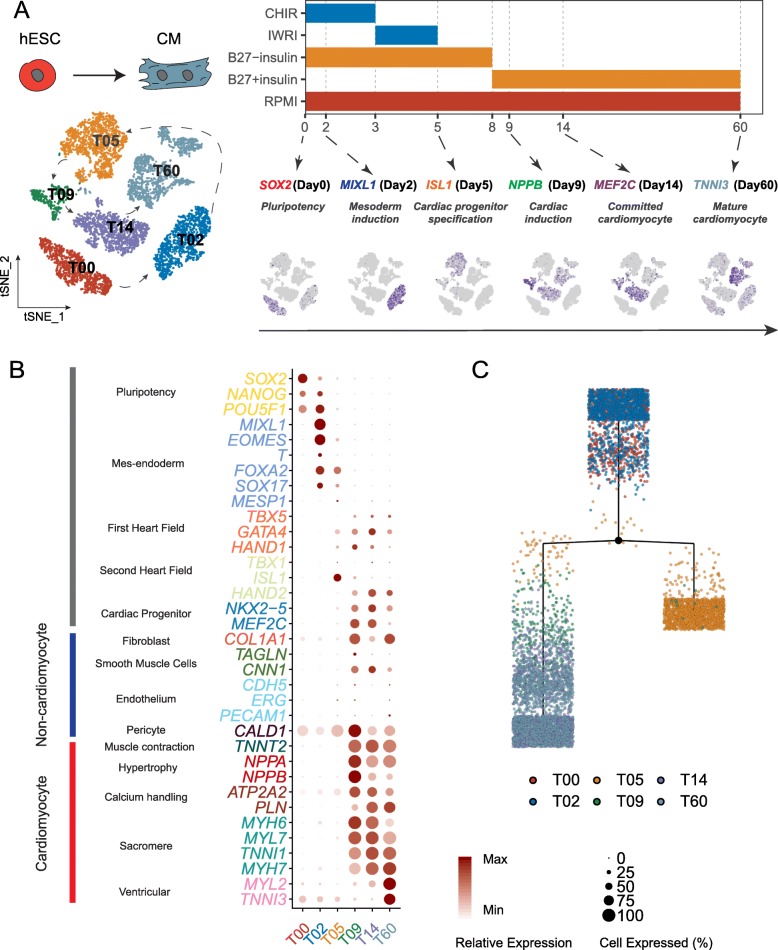
Comprehensive analysis of cardiac differentiation at single-cell resolution. **a** Schematic of the experimental design. Left: t-SNE plot of single-cell clustering from all six time points. Cells are colored by collected time points. Right: CM differentiation protocol (upper panel) and expression of selected markers at specific differentiation stages (lower panel). The shade of indigo color in the t-SNE plot reflects the relative expression level of corresponding genes. **b** The overall expression pattern of canonical markers representing the developmental stages/typical cell types at each time point. The size of the dots reflects the percentage of cells expressing specific marker, and the shade of the dots indicates the relative expression level. **c** Reconstruction of cell differentiation tree using top differentially expressed transcription factors of all time points. The black dot represents the bifurcation point observed on day 5. The end of the branch does not necessarily refer to a developmental end state

To gain insight into the onset of cardiac lineage commitment, we performed scRNA-Seq and obtained data for 6879 cells captured across 6 key time points (days 0, 2, 5, 9, 14, and 60) during in vitro cardiac differentiation of human ESCs (Fig. 1a), using a newly developed single-cell platform, ICELL8 [12]. Here, we applied a widely used cardiomyocyte differentiation protocol [13] that was reported to produce a population of more than 90% cardiac troponin T (*TNNT2*)-positive cardiomyocytes. Cells were sequenced to a median of 2959 genes detected/cell, with a median of 16,903 unique molecular identifiers (UMIs) calculated/cell (Additional file 1: Figure S1A). Non-linear dimension reduction of scRNA-Seq data by t-distributed stochastic neighbor embedding (t-SNE) revealed 6 clearly separated cell populations (T00, T02, T05, T09, T14, T60), each corresponding to a specific time point, indicating the reliability of our platform (Fig. 1a, left panel). The expression of known differentiation stage-specific markers correlated well with each time point/stage (Fig. 1a, right panel). Next, we more closely inspected well-established markers of various stages of differentiation over time (Fig. 1b, Additional file 1: Figure S1B). Pluripotency, mesendoderm, and mesoderm genes were strongly expressed at T00 and T02. Zooming in on these two time points, we identified 3 subpopulations for T00 and 2 for T02 (Additional file 1: Figure S1C). *SOX2* displayed higher expression in T00 subpopulations, especially in T00-C1 and T00-C2, whereas *NANOG* and *OCT4* expression slightly increased from T00 to T02 (Additional file 1: Figure S1D), reflecting their roles in mesoderm commitment. Mesendoderm and mesoderm markers (*MIXL1*, *EOMES*, *T*, *FOXA2*, and *SOX17*), though, were confined to T02 (Additional file 1: Figure S1D). On the contrary, all genes related to cardiac lineage specification did not strongly express until T09, consistent with studies that utilized a separate differentiation protocol [1] (Fig. 1b). Of note, on day 60 of in vitro differentiation, markers of ventricular cardiomyocytes were more strongly expressed, which is in accordance with previous reports that cardiomyocytes differentiated in culture consisted mostly of ventricular-like cardiomyocytes [4] (Fig. 1b). Compared with all other time points, T05 exhibited relatively lower expression of known makers along the differentiation trajectory, with the exception of a small fraction of cells strongly expressed *ISL1*, a transcription factor that marks second heart field progenitors [14] and serves as a marker of cardiac precursors during differentiation (Fig. 1a, b), consistent with a recent report that *ISL1* plays a pioneer role in the epigenetic control of CM cell fate [15]. The lack of apparent molecular signatures in cells at T05 led us to propose that T05 may represent a primed stage key to cardiac lineage commitment. Since transcription factors are known to play determinant roles in cell fate [16, 17], we constructed a differential tree based on differentially expressed transcription factors (*n* = 1253) across 6 time points using embedded tree construction functions of Monocle to further assess this unique transitioning point. Intriguingly, a branching point, instead of a linear trajectory, emerged at T05, suggesting a critical turning point at T05 in cardiac lineage commitment (Fig. 1c).

### Reconstruction of developmental trajectory of cardiac differentiation from human embryonic stem cells

**Fig. 2:**
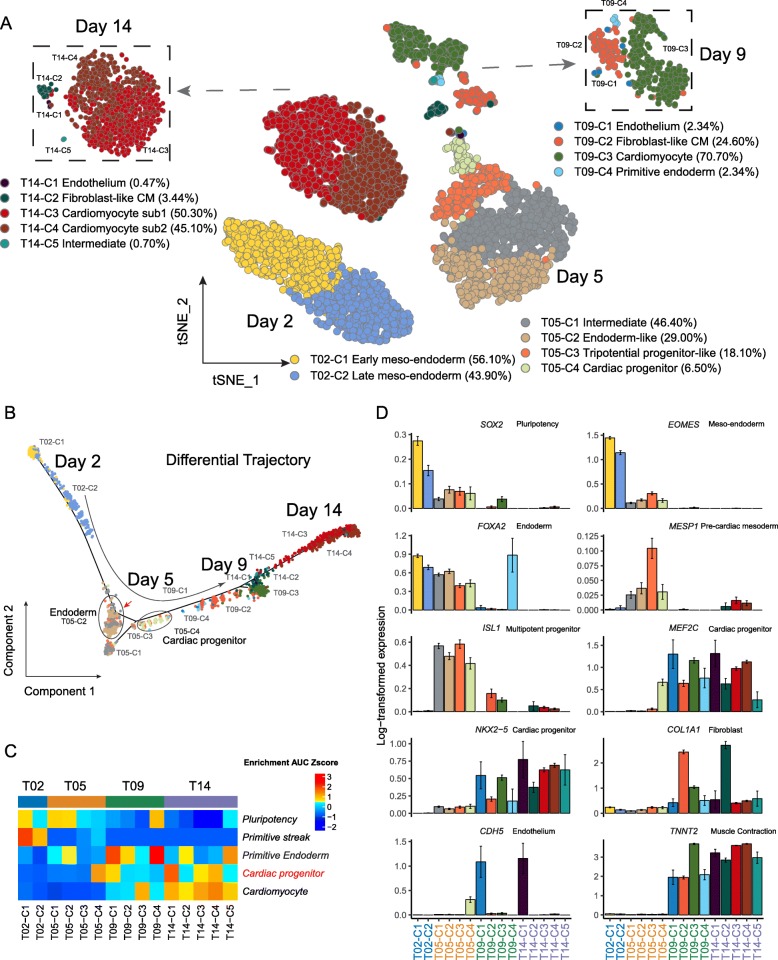
Reconstruction of the developmental trajectory of cardiac differentiation from human embryonic stem cells. **a** A two-dimensional t-SNE plot at a higher resolution displaying the cellular heterogeneity across four time points around cardiac lineage commitment. The subpopulations at day 9 (upper left) and day 14 (upper right) are highlighted separately. **b** Reconstruction of cell differentiation trajectory from day 2 to day 14. Endoderm cells and cardiac progenitors are highlighted on the pseudotime trajectory. The red arrow indicates the bifurcation point. **c** Scaled enrichment area under the curve (AUC) score of known marker genes representing developmental stages/typical cell types across four time points. **d** Barplots to show gene expression of the canonical markers representing various developmental stages/typical cell types across four time points. The height of the bar stands for the mean expression level. The error bar stands for one standard error of mean (SEM)

To more thoroughly characterize the underlying molecular changes surrounding cardiac lineage specification, we focused on the analysis of time points around T05, excluding two extremes (T00 and T60) (Fig. 2a, Additional file 1: Figure S2A). Using the Louvain clustering algorithm, we identified multiple subclusters of T02, T05, T09, and T14 cells (Fig. 2a, b). We further identified these subclusters using markers and normalized gene set enrichment level of known hand-curated human markers (see the “Methods” section). T02 cells were composed of two major subpopulations, early and late mesendoderm cells (Fig. 2a, b), and expressed relatively high levels of mesendoderm marker *EOMES* (Fig. 2d, Additional file 1: Figure S2B). By contrast, T05 consisted of four subpopulations, including intermediate cells (T05-C1), endoderm-like cells (T05-C2), tripotential progenitor-like cells (T05-C3) [18], and cardiac progenitor cells (T05-C4), based on their distinct molecular signatures (Fig. 2a, c, d; Additional file 1: Figure S2B). These observations indicated day 5 as a pivotal time point in the emergence of the cardiac lineage. The appearance of T05-C3, which are *PDGFRA*+*KDR*−, was in accordance with previous studies, demonstrating the reliability of our protocol [1, 19]. By day 9, 70.7% of cells were already committed to the cardiac lineage (T09-C3), marked by elevated expression of cardiomyocyte marker *TNNT2* (Fig. 2d). This proportion of cardiomyocytes further increased to 95.4% on day 14, when cardiomyocytes began to separate into two subclusters based on their expression profiles (T14-C3 50.30% and T14-C4 45.10%) (Fig. 2a, c, d). Reconstruction of the developmental trajectory in pseudotime using Monocle2 showed populations proceeding gradually from mesodermal progenitors to committed and definitive cardiomyocytes, with clear branching and turning at day 5 (Fig. 2b, Additional file 1: Figure S2A). To understand the potential biological functions of the subpopulations, we queried Gene Ontology databases and demonstrated stage-specific enrichment of biological processes related to cardiac differentiation (Additional file 1: Figure S2C, Additional file 2). While T02 populations were linked to early developmental processes such as gastrulation and mesoderm development, T05 clusters showed strong ties to the onset of lineage commitment. For example, tripotential progenitor-like cells (T05-C3) were significantly enriched for nuclear transport (Additional file 1: Figure S2C, Additional file 2), which may be necessary for nuclear import of cardiogenic transcription factors to ensure commitment to a cardiac progeny [20]. In cardiac progenitors (T05-C4), Ras protein signal transduction arose as a major biological event, implying requirement of Ras signals for the specification of heart progenitors [21]. Endoderm cells (T05-C2), by contrast, exhibited marked enrichment for fibroblast growth factor receptor (FGFR) signaling, in line with reports on the importance of FGFR in endoderm development [22]. From T09 onward, cells were apparently committed to a cardiac lineage, marked by a variety of processes pertinent to muscle cell differentiation. Similar trends of functional enrichment were also observed upon KEGG analysis (Additional file 1: Figure S2D, Additional file 2). The expression of individual developmental markers corroborated the presence of distinct subpopulations within T05 cells (Fig. 2c, Additional file 1: Figure S2B). Compared to other T05 subpopulations, endoderm cells (T05-C2) showed increased expression of *FOXA2* (Fig. 2d) and *SOX17* (Additional file 1: Figure S2B) as well as other endoderm markers (Fig. 2c). The endoderm signatures carried on to be enriched in small subclusters on day 9 and day 14 (Fig. 2c). Cardiac progenitors (T05-C4), however, abundantly expressed cardiac progenitor marker *MEF2C*, but lacked endothelial marker *CDH5*, compared to other contemporary cells (Fig. 2c, Additional file 1: Figure S2B). In order to validate the existence of deduced subpopulation, we checked another batch of scRNA-Seq collected on day 5 of hiPSC-CM differentiation with similar experimental protocol [4]. The clustering and expression pattern is highly similar to our observation (Additional file 1: Figure S2E). We further performed immunostaining against APOA2, FOXA2, MESP1, and MEF2C, corresponding markers for T05-C1, T05-C2, T05-C3, and T05-C4 based on single-cell analysis, at day 5, respectively (Additional file 1: Figure S3). As anticipated, APOA2+, FOXA2+, MESP1+, and MEF2C+ cells were observed at day 5 after cardiac differentiation, suggesting the physical presence of these subpopulations. Collectively, our data indicate bifurcation of cells into a cardiac-committed lineage and an endoderm population.

### Crosstalk between endoderm cells and cardiac progenitors potentially regulates cardiac lineage commitment

**Fig. 3:**
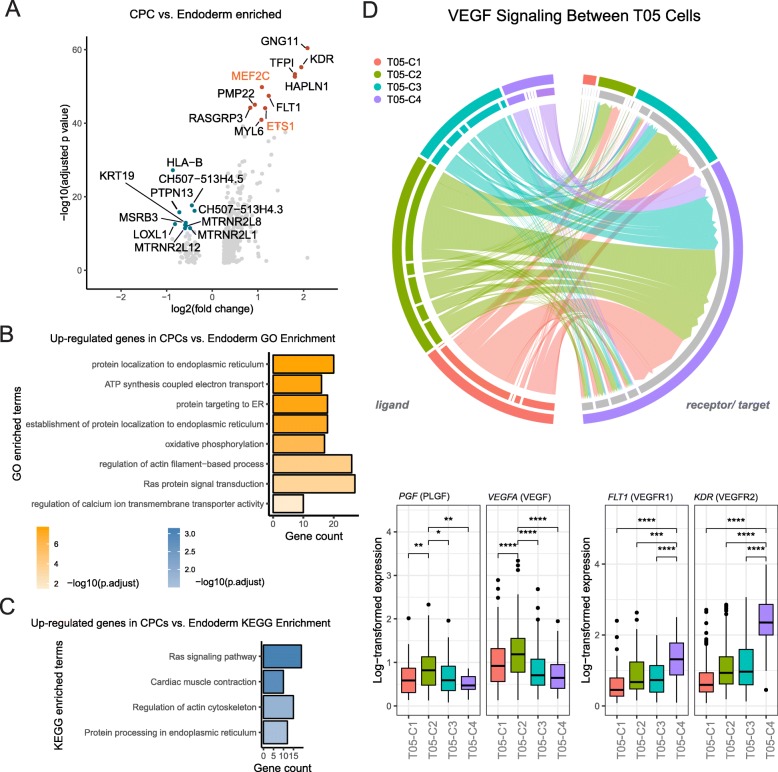
Crosstalk between endoderm cells and cardiac progenitors potentially regulates cardiac lineage commitment. **a** Volcano plot shows the top ten differentially expressed genes (in colored dots) in cardiac progenitor cells (red dots) versus endoderm cells (blue). Transcription factors are highlighted in orange. GO (**b**) and KEGG (**c**) enrichment of differentially expressed genes in cardiac progenitor cells versus endoderm-like cells. The top categories are shown here. Please see Additional file 2 for the full list. **d** Visualization of VEGF signaling among T05 cells (upper panel) using enriched VEGF ligand-receptor pairs (lower panel). The lines indicate the probabilities of a signal being passed between cells

We next sought to determine the functional implications of such divergence and its possible role in cardiac lineage commitment. Analysis of differentially expressed genes (DEGs) between the cardiac progenitor (T05-C4) and the endoderm (T05-C2) populations revealed cardiac progenitor markers *MEF2C* and *KDR* (kinase insert domain protein receptor, also known as *FLK1*) [23] among the top ten enriched genes its designated population (Fig. 3a, Additional file 1: Figure S4A). The upregulation of *MEF2C* and *KDR* can also be observed in day 5 data of hiPSC-CM (Additional file 1: Figure S4B) [4], suggesting our observation is robust. Endoderm cells, however, did not express noticeable markers. GO and KEGG enrichment analyses of upregulated genes in cardiac progenitors demonstrated significant involvement in electron transport, Ras signaling, and cytoskeletal arrangements (Fig. 3b, c), all of which are signs of emerging CMs (Additional file 3). It is well established that in vivo crosstalk between germ layers is essential in determining cardiac cell fate [24, 25]. Additionally, it has been demonstrated in vitro that endodermal cells support cardiac lineage commitment in a paracrine fashion [26, 27]. Therefore, we assembled in silico ligand-receptor interaction pairs to determine whether such crosstalk potentially exists between the T05 subpopulations. The comparisons between T05 subpopulation enriched marker genes and ligand-receptor pairs show that endoderm population could be the main resource to cardiac progenitor cells in potential cell-cell communications (Additional file 1: Figure S4C). Intriguingly, we uncovered that *VEGF*, one significantly upregulated gene in T05-C2 (Fig. 3d), encodes a ligand protein vascular endothelial growth factor A (VEGFA) that binds to vascular endothelial growth factor receptor 1 (*VEGFR1*, also known as *FLT1*), which is one of the top ten upregulated genes in the T05-C4 population (Fig. 3a). Further analysis of all possible ligand-receptor pairs from the two subpopulations unveiled many more potential paracrine routes by which the endoderm population regulates signaling in cardiac progenitor cells. For example, placental growth factor (PGF) is enriched in the endoderm population. PGF is the ligand for VEGF family receptors *FLT1* and *KDR*, which were both enriched in cardiac progenitor cells (Fig. 3d). Similar VEGF family ligand-receptor interaction was also predicted for endoderm cells and tripotential progenitors, but with much lower significance due to the relative low expression of VEGFRs in the latter (Fig. 3d). Thus, it is possible that endoderm cells express competing ligands to tightly regulate signaling in cardiac progenitors.

### ETS1 as an important downstream factor of cell-cell interaction regulating cardiac lineage commitment

**Fig. 4:**
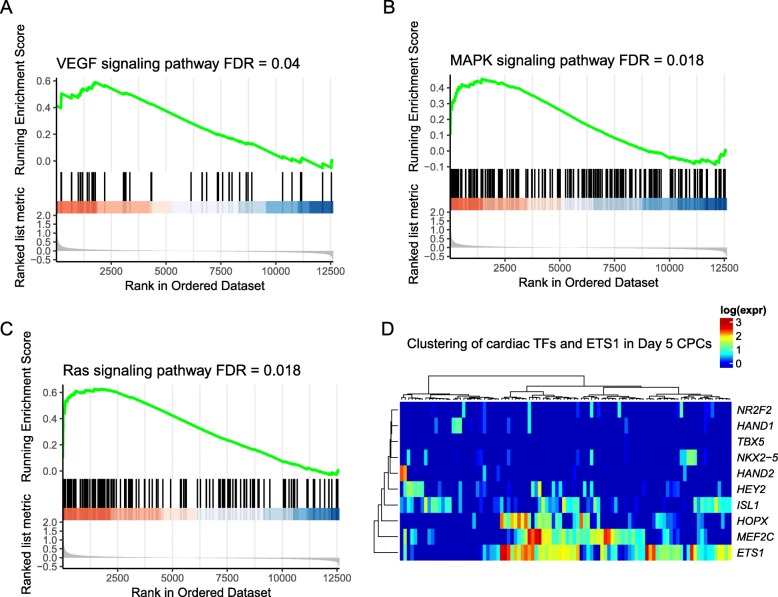
ETS1 as an important downstream factor of cell-cell crosstalk regulating cardiac lineage commitment. **a**–**c** Gene set enrichment analysis (GSEA) shows significant enrichment of three essential pathways potentially activating *MEF2C* and *ETS1* at day 5 in cardiac progenitors. **d** Heat map showing the expression of previously reported cardiac TFs and ETS1 at day 5 in cardiac progenitor population

After observing potential cell-cell crosstalk between endoderm cells and cardiac progenitors, one might wonder to what extent these interactions contribute to cardiac lineage commitment. To this end, we analyzed the downstream signaling pathways elicited in cardiac progenitors associated with cell-cell crosstalk. Consistently, gene set enrichment analysis (GSEA) demonstrated that genes highly expressed in cardiac progenitor cells were dramatically enriched in genes involved in VEGF, MAPK, and Ras signaling pathways, suggesting that activation of these signaling pathways was potentially involved in cardiac lineage commitment (Fig. 4a–c). Noteworthy, these signaling pathways have been suggested to induce *MEF2C* and *ETS1* expression [28–32], both of which were significantly highly expressed in cardiac progenitors compared to other cell subpopulations at T05 (Fig. 3a). Interestingly, when we assessed the expression of several transcription factors that were reported to govern early cardiac fate commitment, as well as cardiac maturation later in the process [2, 4, 33], we observed considerable expression of *ETS1* in cardiac progenitors on day 5, and was significantly co-expressed with *MEF2C* (Rs = 0.26, *p* = 0.01) (Fig. 4d). This upregulation of *ETS1* and *MEF2C* could also be observed in a small population of CPCs from hiPSC-CM differentiation [4], suggesting the robustness of the pattern (Additional file 1: Figure S4B). *MEF2C* is a well-established core regulator of cardiac morphogenesis and myogenesis [34], whereas ETS family transcription factors are largely involved in the commitment of hematopoietic [35, 36] and endothelial lineages [37], with known expression in the cardiac neural crest and heart mesoderm [38–40]. We therefore proposed that the endoderm cell population may act as a supporting cellular microenvironment facilitating cardiac lineage commitment, and insights from this crosstalk may provide clues to identify novel regulators in this process.

### ETS1 highly correlates with cardiac differentiation

**Fig. 5:**
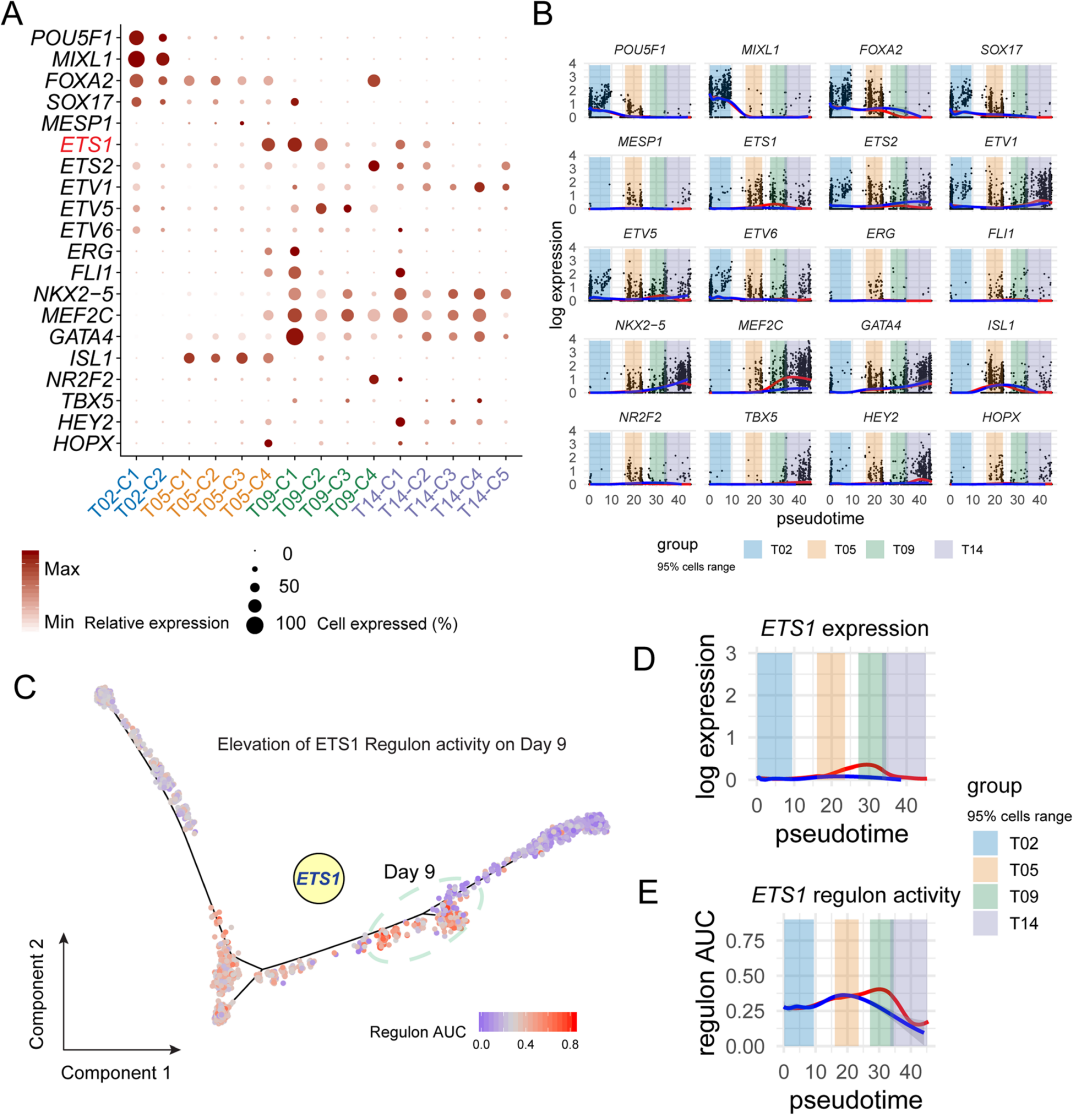
ETS1 highly correlates with cardiac differentiation. **a** The expression pattern of ETS family and other well-characterized cardiac transcription factors in subpopulations from four time points. The size of the dots reflects the percentage of cells expressing specific markers, and the shade of the dots represents their relative expression level. **b** Expression dynamics of well-characterized transcription factors and ETS family along inferred pseudotime. The pseudo-temporal positioning of every cell was based on the estimation when constructing the cell differentiation trajectory in Fig. 2b. The red branch indicates cardiac lineage, and the blue branch indicates endoderm lineage. The branch lines were smoothed using “Loess” function on cells’ expression. The colored regions mark the inferred pseudotime range of 95% of cells from a specific time point. **c** Putative regulon activity (AUC) of EST1 mapped on the differentiation path. The elevation of regulon activity was observed from day 5 to day 9. Day 9 cells are highlighted. Dynamics of expression (**d**) and putative regulon activity (**e**) of ETS1 aligned on differentiation pseudotime

To test our hypothesis, we focused on the putative downstream target ETS1 which was predicted to be activated by cell-cell crosstalk at T05. When comparing all time points, ETS1 was expressed at high levels in T05-C4 (cardiac progenitors) and in T09-C1 (endothelium) and T09-C2 (fibroblast-like CM) populations, as well as in corresponding subpopulations of day 14, albeit at significantly lower levels (Fig. 5a). By contrast, cardiac transcription factors, such as *ISL1* and *MEF2C*, were highly expressed at either early (T05) time points or later stages (T09-T14), respectively, suggesting a shift in the regulation pattern of cardiac differentiation. When plotted in a pseudotime, the expression of *ETS1* also displayed a unique pattern that was distinct from most known cardiac transcription factors as well as members from ETS gene family that have been known to be abundantly expressed in early embryonic heart (Fig. 5b) [41], suggesting ETS1 as a crucial factor in promoting cardiac lineage commitment. Since cardiac differentiation is orchestrated by a sophisticated network of transcription factors, which interact with their cofactors in a unit (i.e., regulon) to regulate each other as well as their effectors [42, 43], we set out to further characterize the temporal dynamics of ETS1 activity during cardiac lineage commitment and differentiation, by applying a computational method specifically designed for scRNA-Seq data [44] to impute highly confident ETS1 regulon and its temporal activities. ETS1 regulon activity remained low on day 2, increased by day 5, and peaked at day 9, after which, it dropped abruptly, which resembled its expression (Fig. 5c–e). Collectively, these data indicate that ETS1 could be an important transcription factor regulating cardiac lineage commitment.

### ETS1 directly regulates cardiac genes to promote cardiac differentiation

**Fig. 6:**
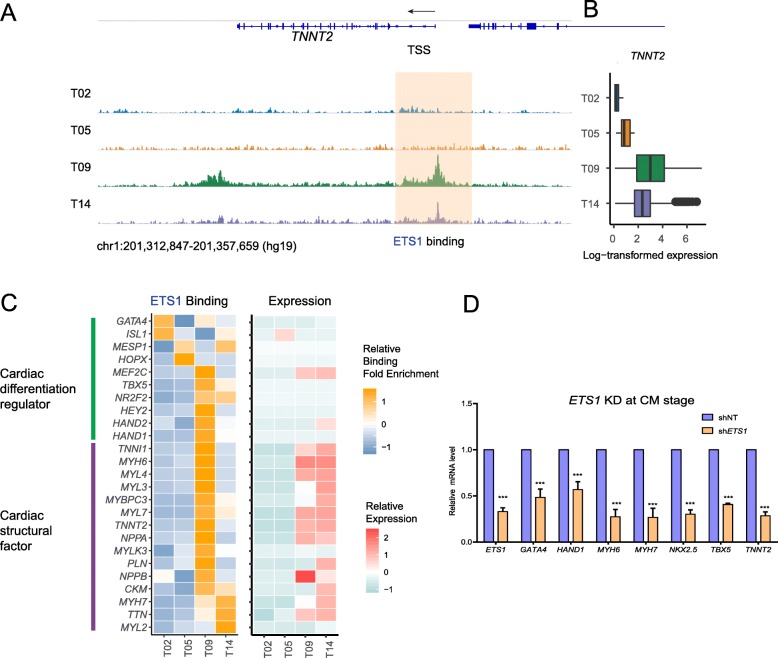
ETS1 directly regulates cardiac genes to promote cardiac differentiation. **a** Genome tracks to show ETS1 occupancy at the transcription start site of TNNT2 at different time points. **b** Boxplot to display the expression level of TNNT2 at corresponding time points. **c** Left: the normalized ChIP-Seq binding signals of ETS1 at the transcription start site of known cardiac TFs and cardiac structural genes. Right: the expression level of the same genes. **d** Real-time PCR to show the expression of cardiac genes in the presence of shNT or sh*ETS1*. Values were normalized to β-actin and plotted as mean ± SEM from three independent experiments. *p* value was calculated by Student’s *t* test. ***p < 0.001

To further unbiasedly evaluate the regulatory potential of ETS1, we assessed its abundance and genomic occupation on days 0, 2, 5, 9, and 14 via chromatin immunoprecipitation followed by high-throughput sequencing (ChIP-Seq). Although *ETS1* is only highly expressed in a small fraction of CPCs on day 5, Western blot analysis shows that the abundance of ETS1 protein is high on day 5 (Additional file 1: Figure S5C). The genomic occupancy of ETS1 remained in the vicinity of transcription start sites from day 1 to day 14 (Additional file 1: Figure S5A), implying the genomic regulatory capacity of ETS1 during the early days of cardiac differentiation. GO enrichment analysis of genes with ETS1 binding signals revealed stage-specific shifts in its gene targets (Additional file 1: Figure S5B, Additional file 4), moving from Wnt signaling and histone modification (important events in cardiogenesis) to axon guidance and then to cytokinesis and cardiac morphogenesis. Specifically, binding of ETS1 to cardiac marker gene *TNNT2*, for example, exhibited pronounced increase on day 9, which declined by day 14, a pattern similar to that of the expression of *TNNT2* (Fig. 6a, b), suggesting an active regulation of *TNNT2* by ETS1 at an early developmental stage. Globally, when examining gene expression and ETS1 binding of cardiac transcription factors that regulate differentiation, and those of cardiac structural genes, we found highly correlated expression levels with ETS1 occupancy on these two groups of genes, suggesting a direct role of ETS1 in activating expression of these genes (Fig. 6c). To substantiate our proposition, we performed shRNA-mediated knockdown of *ETS1* using lentivirus and found dramatic suppression of cardiac genes, including *MYH7*, *MYL3*, *NKX2.5*, *TBX5*, and *TNNT2* (Fig. 6d, Additional file 1: Figure S5D), suggesting an essential role of ETS1 in cardiac lineage commitment.

## Discussion

The in vitro hESC-to-CM differentiation system has been a powerful tool to model human heart development. The rapid advancement of the scRNA-Seq technology enabled a high-resolution representation of gene expression dynamic during differentiation. Combining it with the state-of-art computational technologies, biological curation, and annotations, we were able to dissect the heterogeneity of cells from a single sampling point, as well as to reconstruct a trajectory from multiple sampling points across several developmental stages.

In this study, we focused on the early stages of cardiac cell fate commitment to uncover dynamically changing cell heterogeneity and important regulatory mechanism governing this process. In the past years, several protocols have been developed to obtain a relatively homogeneous CM population. For instance, one of the recent efficient monolayer-based protocols is through modulating canonical Wnt/β-catenin signaling using small molecules on hESCs at early stages [13, 33]. These highly efficient protocols hint at a homogeneous differentiation process throughout the early-middle stage of cardiac differentiation. However, using scRNA-Seq, we found that cardiac progenitor cells (CPCs) emerged alongside several side populations and showed distinct bifurcation paths on the inferred developmental trajectory at the pivotal time junction of cardiac cell fate commitment. By analyzing enriched ligand-receptor pairs in the subpopulations, we unexpectedly found that endoderm cells, one of the side populations at day 5, were deduced to function as a supporting cell population for the development of CPCs. The interaction between endoderm and mesoderm that governs the lineage specification is well known in vivo. Here, we provided evidence that this supporting model still exists in in vitro cardiac differentiation. Further experiments may be required to rank the importance of signaling pathways and ligands utilized to support cardiac lineage commitment in cell-cell interactions.

By using computational analysis of gene regulatory network and performing a loss-of-function experiment, we revealed that transcription factor ETS1 plays an essential regulatory role in mediating early CM development. We have found that both *MEF2C* and *ETS1* were highly enriched in the CPC population and showed a significant level of co-expression with each other, which later assumed different regulatory roles in CM populations and side populations, respectively. In addition, we observed that the Ras signaling pathway was enriched in the CPC population. Interestingly, ETS1, but no other members of the ETS family, was specifically required for the migration of RAS/ERK-activated endothelial cells. These observations further indicated that cellular crosstalk between CPCs and the endoderm cell subpopulation may have activated the Ras signaling pathway in CPCs, the latter of which may further induce ETS1 expression in CPCs. Given the existing knowledge of crosstalk between endothelial cells and cardiomyocytes [45], and that a recent study demonstrated a favorable effect on the maturity of hESC-derived CMs by co-culturing them with endothelial cells [46], we proposed that ETS1 is an important factor in determining early CM fate, possibly through interaction between progenitors of CMs and side cells. These findings warrant a deeper dissection of the crosstalk between the endothelial/endoderm and cardiac lineage, which will further our understanding of heart development and diseases.

Several studies have studied the potential role of the ETS family in heart development. For instance, Schachterle et al. have identified members of the ETS family which function as enhancers to promote cardiac transcription factor expression to regulate heart development [41]. Islas et al. found that *ETS2* and *MESP1* can work together to generate cardiac progenitors de novo from fibroblasts [39]. In our scRNA-Seq based, we identified that *ETS1* was highly expressed in a small portion of cardiac progenitor cells at day 5 and highly expressed in fibroblast-like CM later at day 9. Also, ETS1 exhibited direct binding towards a large set of cardiac structural genes at day 9. The transient expression of *ETS1* suggested its novel regulatory role in early cardiac commitment possibly is required for stabilization of cardiomyogenic fate.

A recent single-cell study of cardiac differentiation from human pluripotent stem cells identified HOP homeobox (*HOPX*) as an important cardiac regulator that could be potentially dysregulated in vitro leads to maturation issues of hESC-CMs [2]. Here, we observed a similar phenotype where *HOPX* was elevated in the CPC population on day 5 and was barely expressed thereafter (Fig. 5a, b). In addition to *HOPX*, we also observed an abrupt elevation in the expression of natriuretic peptide B (*NPPB*), a gene related to cardiac hypertrophy in adult heart, at an early stage of CM differentiation (T09), which dropped quickly thereafter (Fig. 1b). It is known that *NPPB* expression is strongly upregulated in the myocardium at embryonic and fetal stages in vivo [47]. Inspired by these observations, one might wonder whether *NPPB* may be another regulator in cardiac differentiation whose dysregulation could potentially lead to maturation issues of hESC-CMs. Taking it a step forward, it would be useful to identify genes with similar expression patterns as *HOPX* and *NPPB* to help us understand the molecular basis of cardiac maturation both in vivo and in vitro.

## Conclusion

In the study, we not only provided a rich resource and cell-cell crosstalk model supporting cardiac differentiation from hESCs in vitro at single-cell resolution, but also identified ETS1 as a pivotal regulator in early cardiac lineage commitment. These findings may further our understanding of the molecular basis of heart development and provide knowledge for developing novel strategies dictating cardiac differentiation for regeneration purposes.

## Methods

### In vitro hESC culture and differentiation

Human ESC lines (H1) were maintained on Matrigel-coated plates (BD Biosciences) in Essential 8 Medium (STEMCELL Technologies) and ROCK inhibitor (Selleck). The medium was changed every day. For cardiac differentiation, human ESC lines (H1) at ~ 100% confluence were incubated with a differentiation medium comprising RPMI 1640 medium (Gibco) and B27 supplement minus insulin (Invitrogen). On day 0, CHIR99021 (Selleck), a selective glycogen synthase kinase 3β inhibitor, was added to the differentiation medium (3 μM final). On day 3, the Wnt antagonist, IWR-1 (Selleck), was added to the differentiation medium (5 μM final). On day 5, the medium was removed and replaced with a differentiation basal medium without any inhibitors. On day 8, the cells were incubated with a medium consisting of RPMI 1640 medium and B27 supplement plus insulin (Invitrogen). The medium was changed every 3 days for a desired time of culture.

### scRNA-Seq library preparation

The time point of selected 6 different stages are day 0 (hESC), day 2 (MES), day 5 (CP), day 9 (CM), day 14 (immature CM), and day 60 (mature CM). Cells were digested into a single-cell suspension and then filtered through a 70-μm cell strainer into 15-ml tubes. Single-cell sequencing library was generated by iCell8 platform (Takara). In brief, isolated cells were stained with a mixture of Hoechst 33342 and propidium iodide (R37610, Thermo Scientific) according to the manufacturer’s instruction. After staining, cells were washed by PBS and counted by MoxiTM Automated Cell Counter. Afterwards, the cell suspension (20,000 cells/ml) was submitted to the MultiSample NanoDispenser (MSND, Wafergen Biosystems) for single-cell preparation. The dispensed cells were then imaged with the Imaging Station, and single live cells, defined by Hoechst-positive and propidium iodide-negative staining, were selected. Selected cells were subjected to reverse transcription and first-step amplification in a Chip Cycler (Bio-Rad), and the resulting cDNA was purified and size-selected with Agencourt AMPure XP beads (A63880, Beckman Coulter). One nanogram of purified cDNA was applied to generate a sequencing library by using Nextera XT DNA sample preparation Kit (FC-131-1024, Illumina). Libraries were sequenced on the NextSeq500 sequencer (Illumina) using the 26-nt and 50-nt paired-end sequencing protocol.

### scRNA-Seq data bioinformatics pre-processing

Raw sequencing reads were processed in the following four steps: (1) Only read pairs whose read 1 uniquely mapped the pre-defined barcode tag (10 nt) and UMI (14 nt) were considered as valid. (2) Read pairs were filtered by cutadapt (v1.8.1) [48] with the following parameters: -m 20 --trim-n --max-n 0.7 –q20. (3) Reads were then aligned to genomes of human, *Escherichia. coli*, mycoplasma, yeast, and adapter sequences by Bowtie2 (v2.2.4) [49]. Contaminants were filtered by FastQ Screen (v0.5.1.4) [50]. Clean reads were then mapped to UCSC human genome (hg19) via STAR (v2.5.2b) [51] and assigned to Ensembl genes41 by featureCounts [52].

Genes with detected expression (UMI counts ≥ 1) in at most 3 cells will be considered as not expressed and filtered. The human heart is a well-known high-energy tissue, and previous bulk RNA-Seq studies suggested that mitochondrial transcripts comprise almost 30~40% of total mRNA [53, 54]. Here, we observe cells with an expected level of mitochondrial gene expressed along with differentiation (Additional file 1: Figure S1A), so mitochondrial gene expression was not considered as a quality control measure and filtered. Single cells with less than 300 expressed genes were considered as low-quality cells and were filtered. An expression matrix of a total of 7622 single cells was retained after pre-processing.

### scRNA-Seq data clustering and trajectory reconstruction

Seurat R package (v2.3.4) [55] was used to perform further feature selection and clustering. Because of a high dropout rate and stochastic nature of gene expression, single cells were further selected with a number of expressed genes between 1000 (low threshold) and 10,000 (high threshold). A total of 6879 cells were retained for downstream analysis. UMI counts were further normalized among different stages and log-transformed with a default scale factor in Seurat. The highly variable genes (specified in Seurat as outliers on a mean variability plot) were selected with the following parameters: “dispersion.function = LogVMR; x.low.cutoff = 0.0125; x.high.cutoff = 3; y.cutoff = 1.” The confounding factor of expressional variation coming from cell cycle was regressed out using an embedded method in Seurat under the consideration that developmental trajectory could be better inferred by removing the biological covariates (e.g., cell cycle) [56]. Nevertheless, clustering patterns are similar between with and without regression out of cell cycle (Fig. 2a versus Additional file 1: Figure S6A), and there are no significant differences in the composition of cells in different phases among different subclusters (Pearson’ chi-squared test of independence, *p* > 0.05, Additional file 1: Figure S6B). Principal component analysis (PCA) was performed on the expression matrix to capture the eigenvectors. The clustering was using Louvain clustering algorithm on shared nearest neighbor (SNN) as wrapped in “FindCluster” function of Seurat. Cell clusters are defined using the top ten significant principal components under typical resolution “1.0.” t-SNE were then performed after the cell clustering to visualize the results.

The subpopulation marker genes were defined using “FindAllMarkers” function wrapped in Seurat. The Wilcoxon rank-sum test was used with multiple correction. The cutoffs for adjusted *p* value, log fold change, and minimum cell percentage were set as 0.01, 0.25, and 0.05, respectively.

The enrichment level of gene sets corresponded to cell types/development stages were measured by normalized area under the curve (AUC) through AUCell module of the SCENIC [44], and the active regulons were determined by AUCell default threshold. The AUC of each gene set was normalized across all the subpopulations across four time points. The manually curated human single cell (cell types/development stages) markers are gathered from the CellMarker database [57]. Markers from the single-cell dataset were selected in the enrichment.

We performed cell differential trajectory reconstruction by Monocle (v2.6.4) [58] with the top 5000 differentially expressed genes across the subpopulations of 4 time points. To find the top 5000 differentially expressed genes, we used the “differentialGeneTest” function of Monocle and employ the model that finds genes that change as a function of inferred pseudotime. The input of monocle is the raw UMI count. The “DDRTree” algorithm was used in dimension reduction and visualization of the trajectory. The bifurcation tree from 6 time points was constructed on the bases of the cell differential trajectory of all cells using differentially expressed human transcription factor genes (*n* = 1253) across 6 time points.

### Immunostaining

Human CPCs at day 5 were dissociated with 0.25% trypsin (Gibco) and gently scraped off from the wells. Cells were fixed with 4% (vol/vol) paraformaldehyde for 15 min and then permeated and blocked with 0.3% (vol/vol) Triton X-100 and for 5% BSA in goat serum solution for 1 h at room temperature. The samples were then incubated with primary antibody against APOA2 (1:400), FOXA2 (1:400), MESP1 (1:400), and MEF2C (1:400) overnight at 4 °C. Next day, the samples were incubated with secondary fluoresce-labeled anti-mouse/rabbit antibody (1:500) for 1 h at room temperature. The nuclei were stained with DAPI (Invitrogen) for 5 min. Images were captured under Leica sp8 confocal microscopy.

### Gene Ontology, KEGG enrichment, and GSEA analyses

We used R package clusterProfiler (v3.6.0) [59] to perform Gene Ontology enrichment, KEGG enrichment, and GSEA analyses for subpopulation marker genes and differentially expressed genes. Only enriched terms related to cardiac development are visualized in the figure.

### Quantitatively characterizing cell-cell communications

Human transcription factor proteins were extracted from the UniProt database [60]. In total, 1781 genes were retrieved for TF genes. A curated list of human ligand-receptor pairs (*n* = 2557) was retrieved from supplementary files of a previous study [61]. In protein acting information obtained from the STRING database (9606.protein.actions.v10.5), we chose “binding” mode for interacting pairs of ligand-receptors, “activation” and “inhibition” with “combined_score >= 700” as targets as applied in a previous study [61]. The enriched ligand-receptor pairs were retrieved by overlapping the list with marker genes of subpopulations as described in the previous section. The R version of SoptSC [62] was used on our data for calculating the probability matrix of signals being passed between cells and visualization. The enriched ligand-receptor pairs in VEGF signaling and their targets were used to generate Fig. 3d. 

### Gene regulatory network analysis

The analysis of the regulatory network and regulon activity was performed by SCENIC (pyscenic, v0.8.7) [44]. The input to SCENIC is an expression matrix of four time points (day 2, day 5, day 9, and day 14). The MEF2C and ETS1 regulon were imputed using co-expression and binding motifs’ genomic positions specified in the GENIE3 and RcisTarget modules of the SCENIC. The normalized enrichment score (NES) of the transcription factor binding motifs (TFBS) was calculated, and NES > 3.0 was considered as significantly enriched. The gene correlation network was visualized using R package igraph [63]. The regulon activity (measured in AUC) was analyzed by AUCell module of the SCENIC [44], and the active regulons were determined by AUCell default threshold.

### Experimental validation of regulatory rule of ETS by knocking down *ETS1* during cardiac lineage commitment

Human stem cell lines (H1) were seeded onto 6-well plates and transduced with non-targeting (shNT) or *ETS1* (sh*ETS1*) lentivirus-based shRNAs. Cells expressing control or *ETS1* shRNA were selected by puromycin (1 μg/ml) treatment for 2–3 days. Total RNA was extracted from cells using the GeneJet RNA Purification Kit (K0732, Thermo Scientific) 10 days after differentiation. 0.5 μg of total RNA was reverse transcribed to generate cDNA using the iScript cDNA Synthesis Kit (1708891, Bio-Rad) according to the manufacturer’s instructions. qPCRs were performed using the iTaqUniversl SYBR Green supermix (1725121, Bio-Rad) on the Bio-Rad CFX-384 or CFX-96 real-time PCR System. Actin was used for normalization.

### Chromatin immunoprecipitation followed by high-throughput sequencing

Briefly, differentiated cells were fixed using 1% formaldehyde for 10 min, and 0.125 M glycine was added to stop fixation. Cells were harvested, and DNA was fragmented to 300–500 bp by sonication with a Covaris S220 sonicator. Immunoprecipitation was performed with antibodies conjugated to Dynabeads Protein G beads (1004D, Life Technologies). ChIP DNA was eluted, reverse cross-linked, extracted with phenol/chloroform, and precipitated. For ChIP-Seq, 1 ng ChIP DNA or input DNA was used to generate sequencing libraries using the Nextera XT DNA sample preparation Kit (FC-131-1024, Illumina). Libraries were sequenced on the NextSeq500 sequencer (Illumina) using the 35-nt paired-end sequencing protocol.

ChIP-Seq data was aligned to human genome (hg19) by Rsubread (v1.24.2) [64]. Only unique mapped reads were kept. All the bam files were sorted by samtools (v0.1.19-44,428 cd) [65]. Peaks were called by macs2 (v2.1.1.20160309) [66] with parameter “broad.” “BigWig” files were produced by HOMER toolkit [67].

## Supplementary information

**Additional file 1: Figure S1.** Comprehensive analysis of cardiac differentiation at single-cell resolution. **Figure S2.** Reconstruction of developmental trajectory of cardiac differentiation from human embryonic stem cells. **Figure S3.** Immunostaining showing marker genes expression of each subpopulation in the critical transition time point Day 5. **Figure S4.** Crosstalk between endoderm cells and cardiac progenitors potentially regulates cardiac lineage commitment. **Figure S5.** ETS1 directly regulates cardiac genes to promote cardiac differentiation. **Figure S6.** Cell cycle was not a leading factor to distinguish different cell fates at Day.

**Additional file 2.** GO and KEGG enrichment of imputed marker genes of subpopulations from Four time points.

**Additional file 3.** GO and KEGG enrichment of differentially expressed genes in cardiac progenitor cells versus endoderm-like cells.

**Additional file 4.** GO enrichment of genes bound by ETS1 around TSS at indicated time points.

## Data Availability

All relevant data is available from the authors. The processed ChIP-Seq and scRNA-Seq data are deposited under the Gene Expression Omnibus (GEO) with accession number GSE129986 [68] and GSE129987 [69]. The single-cell data were processed and integrated into a data portal (https://hanlab.uth.edu/shiny/hESC2CM/) to visualize the expression and imputed regulon activity.

## Abbreviations

hESC: Human embryonic stem cell
CM: Cardiomyocyte
scRNA-Seq: Single-cell RNA sequencing
ChIP-Seq: Chromatin immunoprecipitation followed by high-throughput sequencing
UMI: Unique molecular identifier
t-SNE: t-distributed stochastic neighbor embedding
CPC: Cardiac progenitor cells
GSEA: Gene set enrichment analysis
MES: Mesoendoderm
AUC: Area under the curve
