# Supplementary material for: Single-cell reconstruction of differentiation trajectory reveals a critical role of ETS1 in human cardiac lineage commitment
Source: BMC Biol. 2019 Nov 13;17:89. doi: 10.1186/s12915-019-0709-6 (PMC6854813; doi:10.1186/s12915-019-0709-6)
Supplement: Supplementary file 1 — Additional file 1: Figure S1. Comprehensive analysis of cardiac differentiation at single-cell resolution. Figure S2. Reconstruction of developmental trajectory of cardiac differentiation from human embryonic stem cells. Figure S3. Immunostaining showing marker genes expression of each subpopulation in the critical transition time point Day 5. Figure S4. Crosstalk between endoderm cells and cardiac progenitors potentially regulates cardiac lineage commitment. Figure S5. ETS1 directly regulates cardiac genes to promote cardiac differentiation. Figure S6. Cell cycle was not a leading factor to distinguish different cell fates at Day. [file 12915_2019_709_MOESM1_ESM.docx]

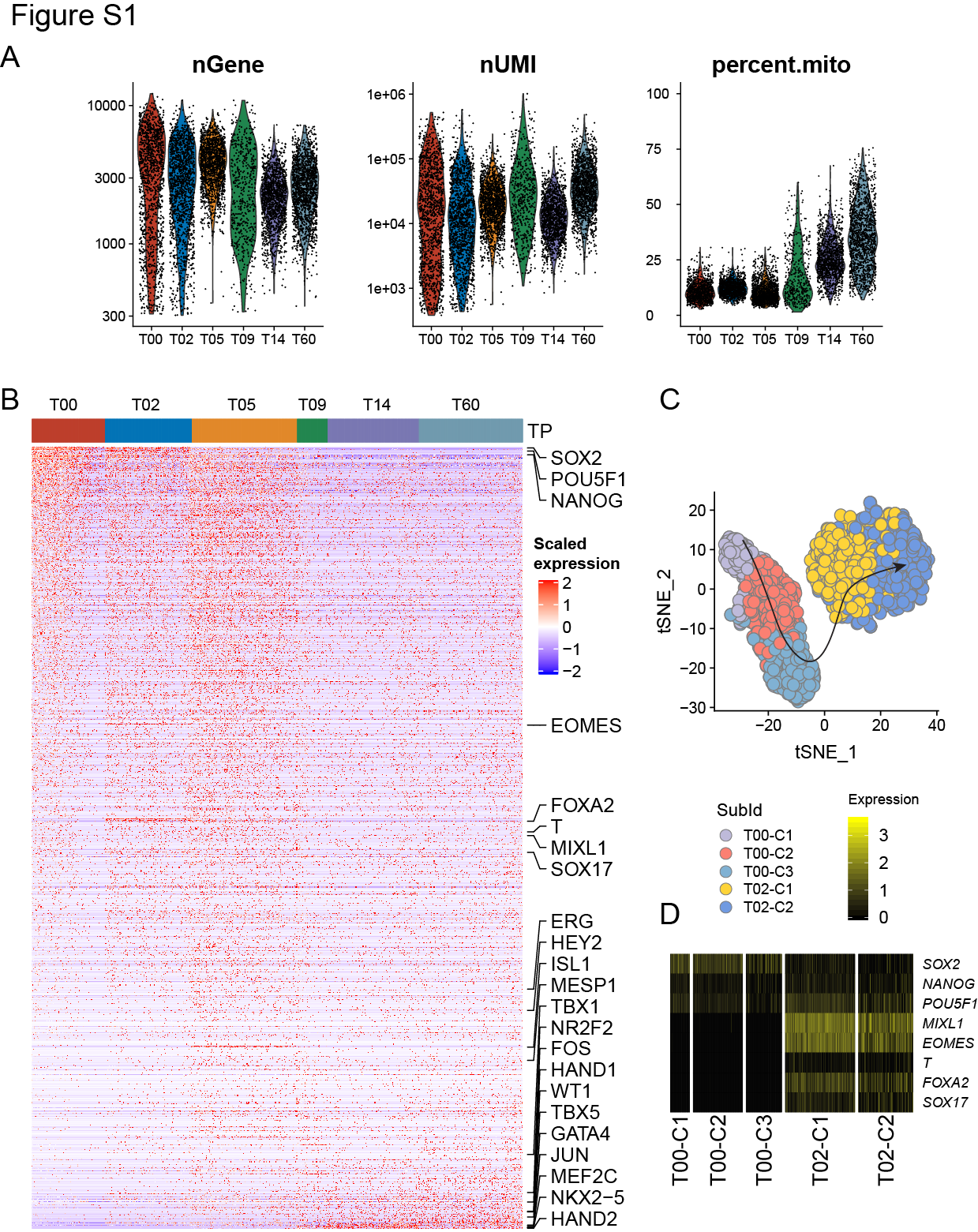


**Figure S1. Comprehensive analysis of cardiac differentiation at single-cell resolution.** (A) Violin plots showing the number of genes detected per cell, number of UMI calculated per cell and percentage of expressed mitochondrial genes per cell across all 6 time points. Y-axises are logarithm scaled for “nGene” and “nUMI”. (B) Heatmap shows the scaled expression levels of differential expressed human transcription factors (n=1253) across six time points. We manually clustered the rows in a way such that the Spearman distances between the TF genes of the top rows are maximized from TF genes of bottom rows. (C) Two-dimensional t-SNE plot of cells collected from the first 2 days of hESC *in vitro* differentiation. (D) Heatmap to show the expression of transcription factors critical for the pluripotent and mesendoderm stages during hESC differentiation.


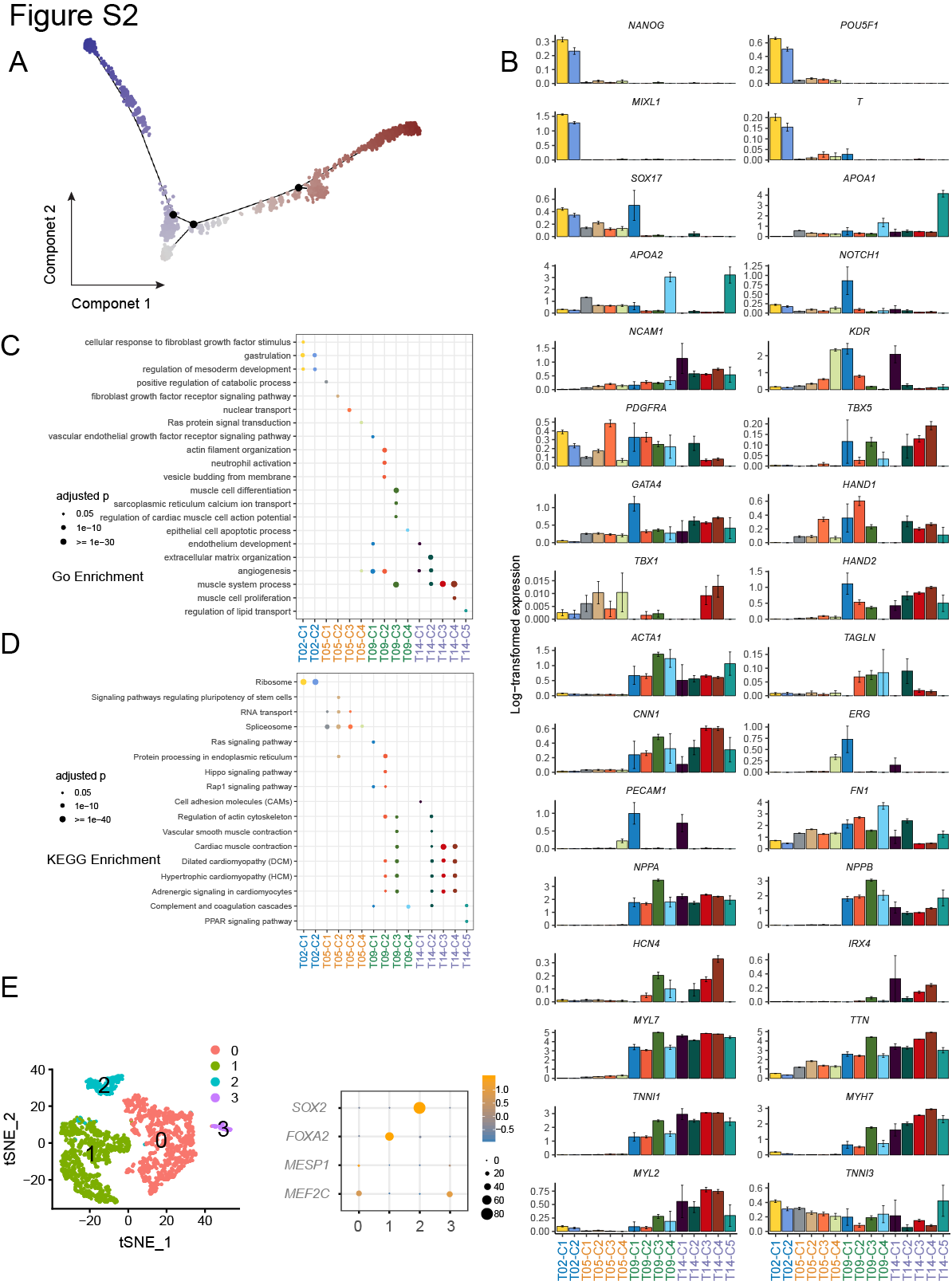


**Figure S2. Reconstruction of developmental trajectory of cardiac differentiation from human embryonic stem cells.** (A) Imputed pseudotime of cells along the differentiation trajectory. (B) Gene expression pattern of the canonical markers representing developmental stages/ typical cell types across subpopulations from four time points. The error bar stands for one SEM. (C-D) GO (C) and KEGG (D) enrichment of imputed marker genes of subpopulations from 4 time points. Selected categories are shown here. Please see table S2 for the full list. (E) Analysis of scRNA-seq data collected from Day 5 of hiPSC-CM differentiation. Left: T-SNE plot suggests four major subpopulations. Right: Expression pattern of cell markers/ cardiac regulators in different subpopulations. The size of dots reflects the percentage of cells expressing specific marker, and the color of dots indicates the relative expression level.


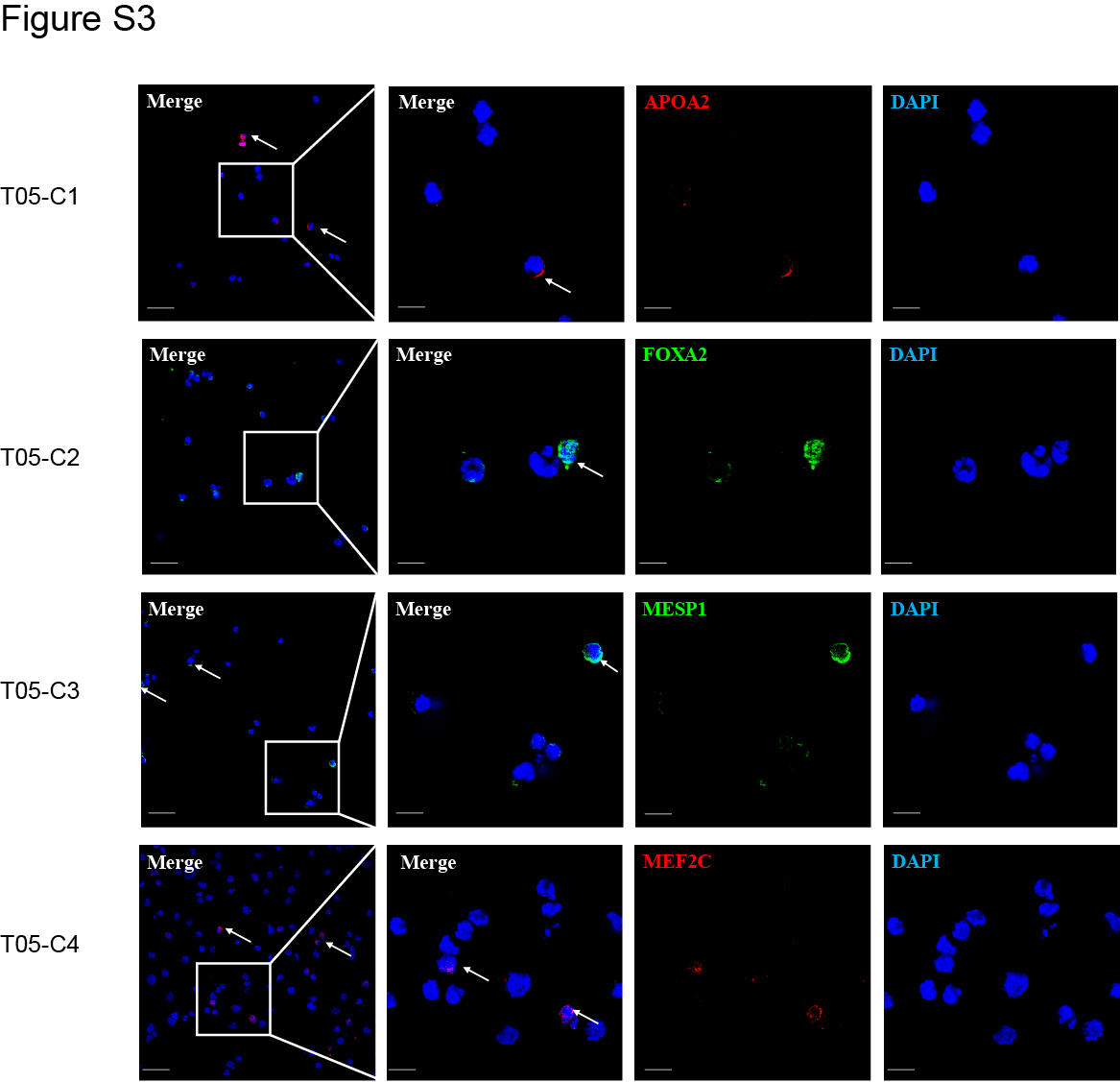


**Figure S3. Immunostaining showing marker genes expression of each subpopulation in the critical transition time point Day 5.** White arrows indicate positive cells, scale bar = 50μm for the figures in the first column, scale bar = 25μm for rest of the figures.


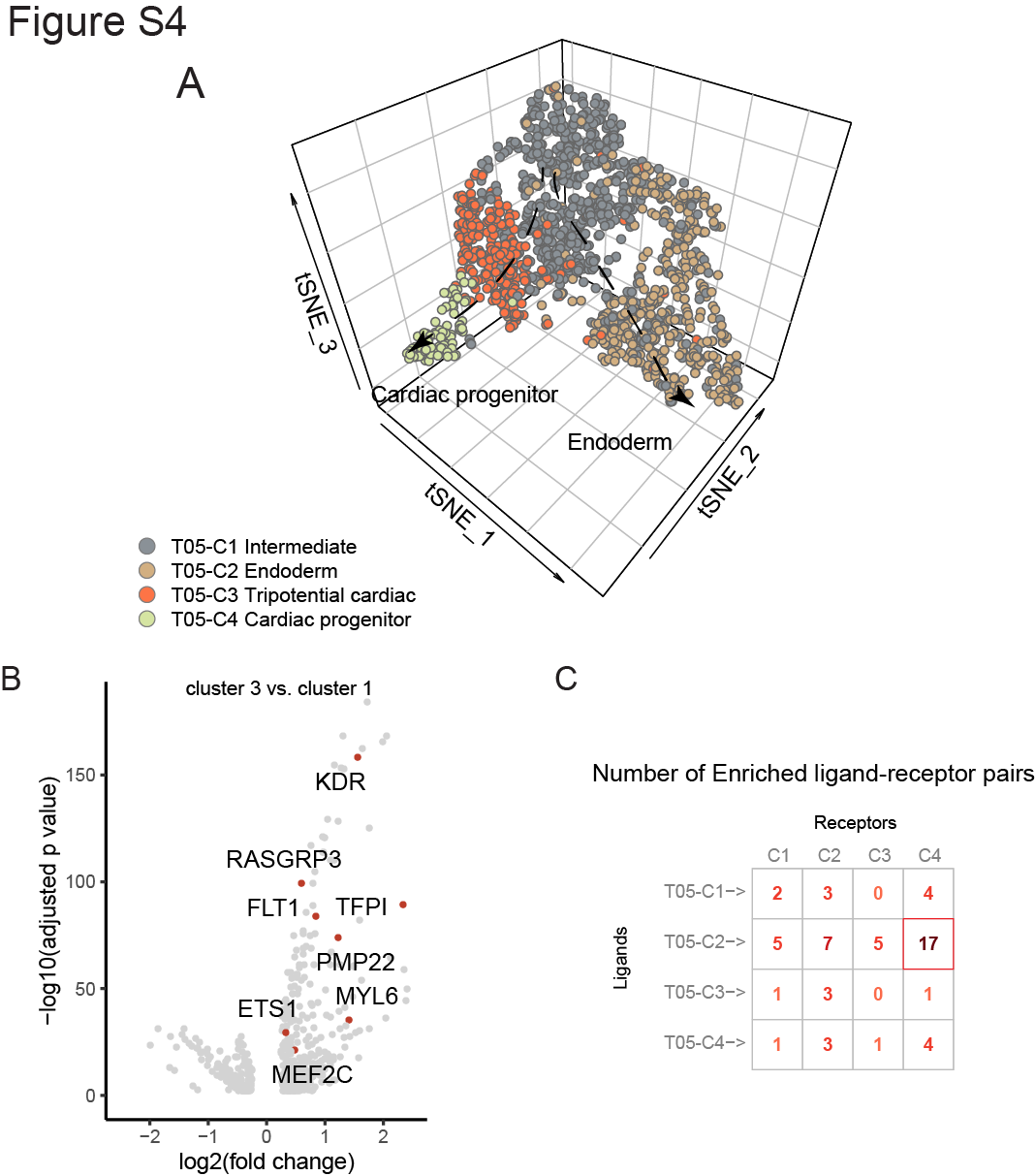


**Figure S4. Crosstalk between endoderm cells and cardiac progenitors potentially regulates cardiac lineage commitment.** (A) A 3-dimensional t-SNE plot to display differentiation path around day 5. (B) Volcano plot shows differentially expressed genes in cardiac progenitor cells (cluster 3) versus endoderm cells (cluster 1). Top 10 upregulated genes in Figure 3A are highlighted in red dots. (C) Numbers of ligand-receptor pairs between four populations on Day 5.


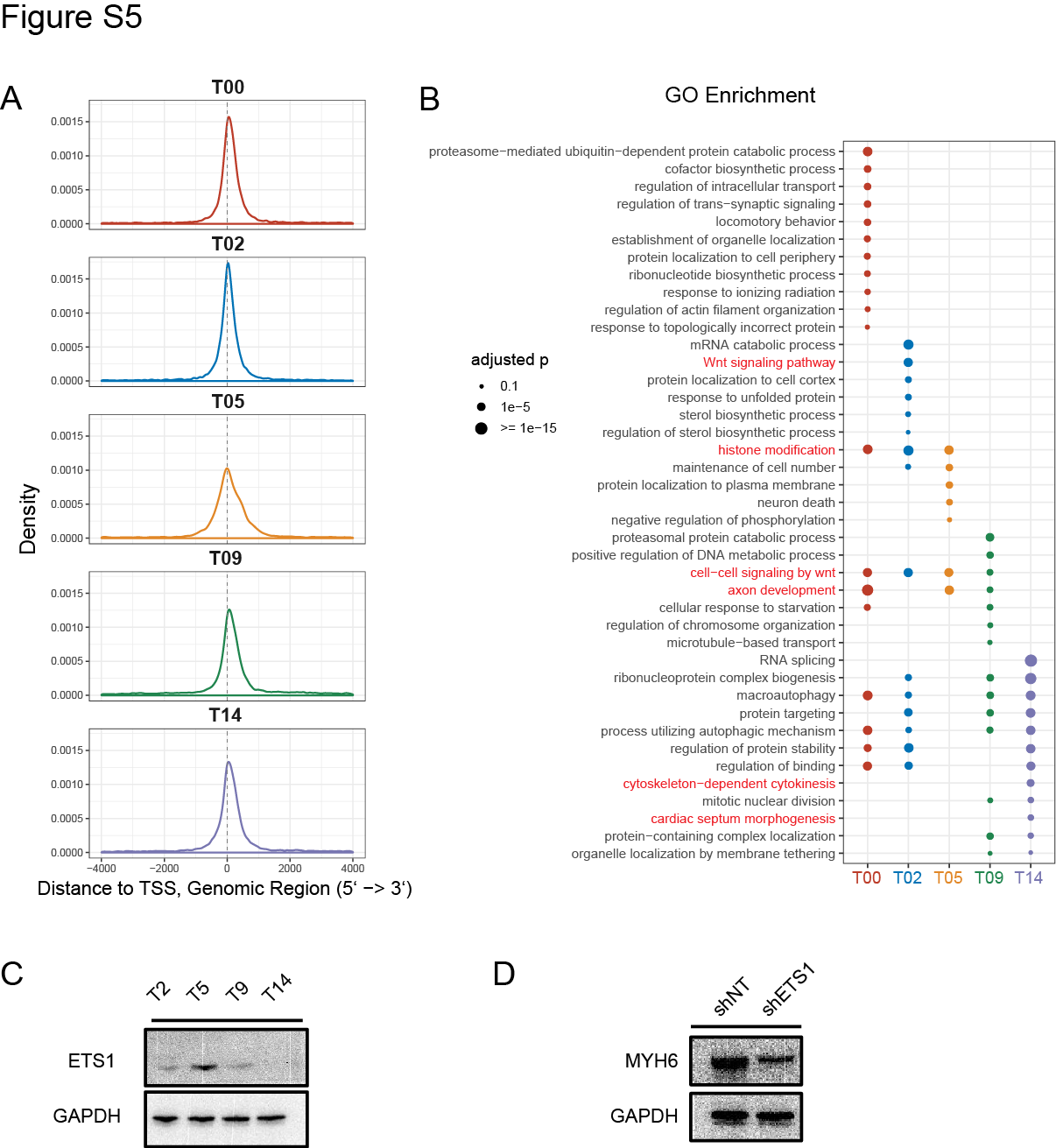


**Figure S5. ETS1 directly regulates cardiac genes to promote cardiac differentiation.** (A) Distribution of ETS1 ChIP-Seq signals around transcription start site (TSS) across different differentiation stages. (B) Gene ontology (GO) enrichment of genes bound by ETS1 around TSS at indicated time points. Please see table S3 for the full list. (C) Western blot analysis demonstrates ETS1 abundance at four time points of CM differentiation. Cell lysates at the indicated differentiation stages. GAPDH was used as loading control. (D) Experimental validation of *MYH6* expression using Western blot upon ETS1 silencing. GAPDH was used as loading control.


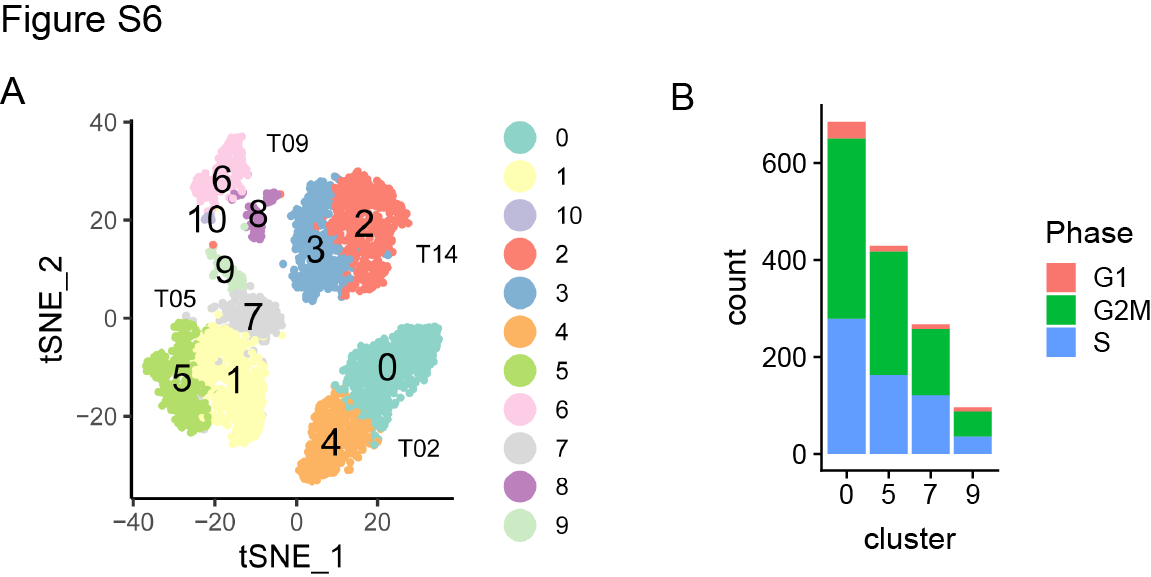


**Figure S6.** **Cell cycle was not a leading factor to distinguish different cell fates at Day 5.** (A) A two-dimensional t-SNE plot displaying the cellular heterogeneity across four time points colored by subclusters under resolution 1. Collected time points are marked. (B) Number of cells in subclusters from Day 5 inferred as characterized in different cell cycles (G1, G2M and S).
